# Communication skills training and the conceptual structure of empathy among medical students

**DOI:** 10.1007/s40037-018-0431-z

**Published:** 2018-04-18

**Authors:** Daisuke Son, Ikuo Shimizu, Hirono Ishikawa, Muneyoshi Aomatsu, Jimmie Leppink

**Affiliations:** 1 International Research Center for Medical Education, Graduate School of Medicine, The University of Tokyo, Tokyo, Japan; 2 Center for Medical Education and Clinical Training, Shinshu University, Matsumoto, Japan; 3 Department of Health Communication, Graduate School of Medicine, The University of Tokyo, Tokyo, Japan; 4 Department of Medical Education, Saku Central Hospital, Saku, Japan; 5 School of Health Professions Education, Maastricht University, Maastricht, The Netherlands

**Keywords:** Communication skills training, Jefferson Scale of Empathy, Medical students, Objective structured clinical examination, Empathic behaviour

## Abstract

**Introduction:**

Medical and healthcare professionals’ empathy for patients is crucially important for patient care. Some studies have suggested that a significant decline in empathy occurs during clinical training years in medical school as documented by self-assessed empathy scales. Moreover, a recent study provided qualitative evidence that communication skills training in an examination context, such as in an objective structured clinical examination, might stimulate perspective taking but inhibit the development of compassionate care. Therefore, the current study examined how perspective taking and compassionate care relate to medical students’ willingness to show empathic behaviour and how these relations may change with communication skills training.

**Methods:**

A total of 295 fourth-year Japanese medical students from three universities completed the Jefferson Empathy Scale and a newly developed set of items on willingness to show empathic behaviour twice after communication skills training, pertaining to post-training and retrospectively for pre-training.

**Results:**

The findings indicate that students’ willingness to show empathic behaviour is much more correlated with perspective taking than with compassionate care. Qualitative descriptive analysis of open-ended question responses revealed a difficulty of feeling compassion despite showing empathic behaviour.

**Discussion:**

These findings shed light on the conceptual structure of empathy among medical students and generate a number of hypotheses for future intervention and longitudinal studies on the relation between communication skills training and empathy.

**Electronic supplementary material:**

The online version of this article (10.1007/s40037-018-0431-z) contains supplementary material, which is available to authorized users.

## What this paper adds

Medical and healthcare professionals’ empathy for patients is crucially important for patient care. Some studies have suggested that a significant decline in empathy occurs during clinical training years in medical school as documented by self-assessed empathy scales. This study indicates that students’ willingness to show empathic behaviour is much more related to perspective taking than to compassionate care.

## Introduction

Physicians’ empathy for patients is crucially important for gathering appropriate information from patients and for understanding patients’ illnesses and suffering. Empathy enables a physician to provide quality patient care [1] without becoming too emotionally involved [2, 3]. Moreover, empathy appears to be associated with personal and professional growth, well-being and career satisfaction [4–6]. Finally, empathy is positively related to patient satisfaction, increased compliance to treatment and fewer complaints of malpractice by patients [7].

Some studies suggest that a significant decline in empathy occurs during the clinical training years in medical school as documented by self-assessed empathy scales along with an increased cynicism and emotional exhaustion in later years [4, 5, 8–10]. However, more recent work underlines that changes in empathy during medical school should not be conceived in terms of some ‘overall’ decline but that different components of empathy have to be assessed to understand where changes take place [7]. Whether or not we detect and come to understand changes in empathy may to some extent be a matter of perspective and definition. If we perceive empathy mainly in terms of understanding of patients’ experiences, concerns and perspectives combined with a capacity to communicate this understanding with an intention to help [11–13], we view empathy through a cognitive lens. Some have distinguished between a *perspective taking* or cognitive component on the one hand and a *compassionate care* or emotive component on the other hand [14, 15]. Others have gone one step further by arguing that compassionate care is an integral part of sympathy rather than of empathy [16, 17].

When we adopt the view that perspective taking and compassionate care are two components of empathy, an instrument that attempts to measure these components is found in the Jefferson Scale of Empathy [10–12, 15]. This instrument was designed to measure empathy in physicians and other healthcare professionals as well as students in medicine and healthcare. It has been translated into more than 50 languages and has been used in more than 80 countries.

When administered together with an instrument that can measure students’ willingness to show empathic behaviour, we may gain understanding of the roles of perspective taking and compassionate care in students’ willingness to show empathic behaviour. If compassionate care has a substantial role, students who are more willing to show empathic behaviour should be inclined to give higher ratings on compassionate care. Consequently, we should be able to establish a positive correlation between these two variables. Moreover, we might see changes in that correlation with communication skills training. On the one hand, if the role of compassionate care in students’ willingness to show empathic behaviour increases with training, we should see a *higher* correlation between compassionate care and willingness to show empathic behaviour after than before training. On the other hand, if the role of compassionate care in students’ willingness to show empathic behaviour is reduced with training, we should see a *lower* correlation between compassionate care and willingness to show empathic behaviour after than before training.

The latter scenario appears to receive support from a cross-sectional cohort study in Japan: empathy scores did not differ substantially across the six years of medical school and focus group sessions revealed that communication skills training in an examination context, such as in an objective structured clinical examination (OSCE), might inhibit the development of compassionate care and stimulate perspective taking instead [18, 19]. In other words, even if compassionate care relates to empathy in the initial stage, it might start doing so to a lesser extent with communication skills training.

Therefore, the current study examined how perspective taking and compassionate care relate to medical students’ willingness to show empathic behaviour and how these relations may change with communication skills training in a regular training and assessment context in a medical curriculum. In line with the findings from the aforementioned cross-sectional cohort study in Japan, we expected that the relation between perspective taking and willingness to show empathic behaviour would increase whereas the relation between compassionate care and willingness to show empathic behaviour would decrease with communication skills training.

## Method

The current study was conducted from October 2015 to February 2016 in medical schools in three Japanese universities.

### Participants and design

A total of 295 fourth-year Japanese medical students from three universities completed the Jefferson Scale of Empathy and a newly developed willingness to show empathic behaviour scale twice after communication skills training, pertaining to post-training and retrospectively for pre-training. In other words, students completed the questionnaire once for post-training and once retrospectively on their empathy before training. While the retrospective pre/post self-report approach comes with the limitations of possible memory effects and participants’ tendency to think that some change—preferably learning—must have taken place (i.e., good subject effect) [20], it has the advantage over an actual pre/post design that it does not suffer from a response shift bias due to a relative inability to assess one’s own ability when prior knowledge or experience is lacking [21]. One of the consequences of such a response shift bias could be that students give lower post-OSCE than pre-OSCE ratings on perspective taking, compassionate care or willingness to show empathic behaviour because the OSCE has made them more aware of how much they can still grow rather than that the OSCE results in an actual decrease in any of these factors.

### Materials and procedure

The OSCE is a common achievement test conducted every year in all medical schools in Japan, usually in the late fourth year in a six-year curriculum, to assess students’ clinical competencies. Most medical schools, including the three medical schools that participated in the current study, teach communication skills to fourth-year students using simulated patients in several medical interviewing training sessions before the OSCE takes place. Questionnaire administration took place right after the OSCE.

We used the Japanese student version (i.e., S‑Version) of the Jefferson Scale of Empathy [18]. This questionnaire has 20 items, each to be rated on a seven-point Likert scale. In previous studies, factor analysis revealed three factors: perspective taking (10 items), compassionate care (8 items) and standing in the patient’s shoes (2 items) [22]. As we were interested in how perspective taking and compassionate care relate to willingness to show empathic behaviour, we developed a new three-item scale that aimed to measure students’ willingness to show empathic behaviour and fit in the context of the training. The training has been a regular part of the medical curriculum in the universities that participated in this study. Face validity of the willingness to show empathic behaviour items was acquired from the experiences and comments from tutors engaged in the training as well as from students. Table 1 presents the entire questionnaire of perspective taking, compassionate care and willingness to show empathic behaviour items.

**Table 1:** The questionnaire used in this study. Assumptions: items 2, 4, 5, 9, 10, 13, 15–17 and 20 are indicators of perspective taking; items 1, 7, 8, 11, 12, 14, 18 and 19 are indicators of compassionate care; and items 21–23 are indicators of willingness to show empathic behaviour

1	I believe that empathy is an important therapeutic factor in medical treatment
2	Patients feel better when their physicians understand their feelings
3	It is difficult for a physician to view things from a patient’s perspectives
4	Understanding body language is as important as verbal communication in physician-patient relationships
5	A physician’s sense of humour contributes to a better clinical outcome
6	Because people are different, it is difficult to see things from the patient’s perspective
7	Attention to patients’ emotions is not important in history taking
8	Attentiveness to patients’ personal experiences does not influence treatment outcomes
9	Physicians should try to stand in their patients’ shoes when providing care to them
10	Patients value a physician’s understanding of their feelings, which is therapeutic in its own right
11	Patients’ illnesses can be cured only by medical or surgical treatment; therefore physicians’ emotional ties with the patients do not have a significant influence in medical or surgical treatment
12	Asking patients about what is happening in their personal lives is not helpful in understanding their physical complaints
13	Physicians should try to understand what is going on in their patients’ minds by paying attention to their non-verbal cues and body language
14	I believe that emotion has no place in the treatment of medical illness
15	Empathy is a therapeutic skill without which the physician’s success is limited
16	Physicians’ understanding of the emotional status of their patients, as well as that of their families, is one important component of the physician-patient relationship
17	Physicians should try to think like their patients in order to render better care
18	Physicians should not allow themselves to be influenced by strong personal bonds between their patients and their family members
19	I do not enjoy reading non-medical literature of the arts
20	I believe that empathy is an important therapeutic factor in medical treatment
21	I will show empathic behaviour to patients when I see their distress
22	I will show empathic behaviour to patients when I see them feeling pain
23	I will show empathic behaviour to patients when I hear their difficult experiences

Additionally, we included one open-ended question: ‘How did medical interviewing training sessions and the OSCE influence your communication skills in terms of empathy?’ Finally, we also asked students to indicate the number of medical interviewing training sessions they had attended.

### Data analysis

Although 295 students participated in the study, seven students (2.4% of 295 students) completed fewer than 53% of the items. Furthermore, of the 288 students who completed the questionnaire (217 men, 66 women, and 5 did not specify; average age 23.1 years, eight students did not specify), one student omitted 13% of the items, one student omitted 11% of the items, 20 other students omitted less than 7% of the items, and the remaining 268 students responded to all items.

To examine the relation between perspective taking (10 items), compassionate care (8 items) and willingness to show empathic behaviour (3 items), we performed confirmatory factor analysis in Jamovi 0.8.1.18 [23] , conform the questionnaire structure displayed in Table 1, with full information maximum likelihood estimation for missing response [24], for both post-training and pre-training, respectively. Moreover, to gain an additional understanding of the relation between the number of medical interviewing training sessions and each of perspective taking, compassionate care and willingness to show empathic behaviour, we performed linear mixed-effects analysis in Jamovi 0.8.1.18 [23] , treating retrospective pre-training and post-training as repeated measurements, the number of medical interviewing training sessions as between-subjects factor and student-level intercept as random effect. The latter was based on only 234 students (79% of 295 students) due to non-response of the other students to the number of medical interviewing training sessions and only one student indicating 0 medical interviewing training sessions. Thus, the number of medical interviewing training sessions consisted of three categories: 1 (*n* = 31), 2 (*n* = 135) and 3 (*n* = 68).

Finally, responses to the open-ended question on the self-perceived influence of the number of medical interviewing training sessions and OSCE on their communication skills in terms of empathy were subjected to qualitative descriptive analysis [25] to check for recurring themes that could help triangulate the findings from confirmatory factor analysis and/or linear mixed-effects analysis. First author DS conducted the initial coding and three other authors (IS, HI and MA) reviewed that coding. Disagreements were discussed until consensus was reached.

### Ethical considerations

Students received an informed consent form, and those who understood the purpose of the research and agreed to participate received the questionnaire. Questionnaires were administered anonymously. Participants were informed that the survey was not mandatory and had no relation to grading or study progress. The study was conducted under the approval of the Institutional Review Board of the University of Tokyo, Shinshu University, and Nagoya University, where the study was implemented.

## Results

Tables 2 and 3 present the outcomes of confirmatory factor analysis of retrospective pre-training and post-training. Additionally, a correlation matrix of the items for each of retrospective pre-training and post-training is included in an Appendix, which can be found online as Electronic Supplementary Material.

**Table 2:** Confirmatory factor analysis for retrospective pre-training (comparative fit index = 0.947; Tucker-Lewis index = 0.940; root mean square error of approximation = 0.057) and post-training (comparative fit index = 0.918; Tucker-Lewis index = 0.908; root mean square error of approximation = 0.068): standardized loadings

Factor	Item	Pre	Post
Perspective taking	2	0.522	0.494
	4	0.631	0.651
	5	0.319	0.319
	9	0.736	0.652
	10	0.467	0.474
	13	0.773	0.732
	15	0.495	0.397
	16	0.799	0.744
	17	0.432	0.404
	20	0.721	0.724
Compassionate care	1	0.499	0.455
	7	0.782	0.714
	8	0.862	0.779
	11	0.794	0.836
	12	0.774	0.756
	14	0.684	0.785
	18	0.183	0.253
	19	0.542	0.580
Willingness to show Empathic behaviour	21	0.958	0.949
	22	0.986	0.973
	23	0.946	0.941

**Table 3:** Confirmatory factor analysis for retrospective pre-training: factor correlations

Factor	Factor	Pre	Post
Perspective taking	Compassionate care	0.494	0.469
Perspective taking	Willingness to show empathic behaviour	0.631	0.699
Compassionate care	Willingness to show empathic behaviour	0.244	0.334

The comparative fit index and Tucker-Lewis index are estimates of the proportion of sample information explained by the model, with a theoretical range of 0–1, and values above 0.90 generally being considered adequate [26]. The root mean square error of approximation should be below 0.08 and preferably below 0.06 [27]. In other words, the comparative fit index values of 0.947 (retrospective pre-training) and 0.918 (post-training) indicate good fit, and the root mean square error of approximation values of 0.057 (retrospective pre-training) and 0.068 (post-training) are also reasonable.

### Perspective taking, compassionate care, willingness to show empathic behaviour interrelations

The high loadings for willingness to show empathic behaviour indicate that the attempt to create a unidimensional willingness to show empathic behaviour scale has succeeded. The correlation between perspective taking and compassionate care was 0.494 for retrospective pre-training and 0.469 for post-training. The correlation between perspective taking and willingness to show empathic behaviour was 0.631 for retrospective pre-training and 0.699 for post-training. The correlation between compassionate care and willingness to show empathic behaviour was 0.244 for retrospective pre-training and 0.334 for post-training.

### How the number of medical interviewing training sessions relates to perspective taking, compassionate care and willingness to show empathic behaviour

Table 4 shows the mean scores and standard deviations of perspective taking, compassionate care and willingness to show empathic behaviour for retrospective pre-training and post-training, and for each number of medical interviewing training sessions (i.e., 1, 2 or 3).

**Table 4:** Means (and standard deviations) for perspective taking, compassionate care and willingness to show empathic behaviour for retrospective pre- and post-training per number of medical interviewing training sessions

Occasion	Medical	Perspective	Compassionate	Willingness to show
	Interviewing training sessions	Taking	Care	Empathic behaviour
Pre	1	5.522 (0.829)	5.372 (0.783)	5.033 (1.108)
	2	5.262 (0.940)	5.097 (0.866)	5.346 (1.294)
	3	5.209 (0.986)	5.380 (0.793)	5.392 (1.173)
Pre	1	5.560 (0.999)	5.679 (0.678)	5.544 (1.030)
	2	5.522 (1.041)	5.548 (0.858)	5.843 (1.163)
	3	5.552 (1.022)	5.740 (0.748)	5.912 (1.167)

Although there was a modest increase in perspective taking (95% confidence interval: 0.057–0.150 points), compassionate care (95% confidence interval: 0.150–0.240) and willingness to show empathic behaviour (95% confidence interval: 0.179–0.333) from retrospective pre-training to post-training, both the main effect of the number of medical interviewing training sessions and its interaction with occasion were small and not statistically significant for any of these factors. In other words, we failed to find evidence for any relation between the number of medical interviewing training sessions and perspective taking, compassionate care or willingness to show empathic behaviour.

### Self-perceived effects of medical interviewing training sessions and OSCE on communication skills with regard to empathy

Table 5 highlights the 16 themes that emerged, with 12 themes on positive aspects of learning and 4 themes on difficulties of learning.

**Table 5:** Qualitative descriptive analysis of students’ open-ended responses

What students learned	Learning how to show empathic attitudes to patients
	Learning the procedure of medical interviewing
	Learning the importance of patients’ perspective taking
	Learning the importance of plain explaining
	Recognizing that communication skill are learnable
	Influence of communication on physician-patient healing relationship
	Learning the importance of listening to patients
	Recognizing that medical interviewing training is a process of socialization
	Variety of patients’ responses to the same question
	Learning the importance of efficacy of communication
	Recognizing that empathy is an essential characteristic of a good physician
	Learning that appropriate spacing is important
What students found difficult to learn	Difficulty of patients’ perspective taking
	Difficulty of empathizing patients’ feelings
	Difficulty of conveying what doctor wants to patients
	Difficulty of feeling compassion despite showing empathic behaviour

On the positive aspects, students reported among others ‘*learning how to show empathic attitudes to patients*’ and ‘*recognizing that communications skills are learnable.*’ Recurring difficulties were the ‘*difficulty of empathizing with patients’ feelings*’ and the ‘*difficulty of feeling compassion despite showing empathic behaviour*’. Some students elaborated further. For instance, one student proceeded that ‘*even if I try to show compassion, sometimes I cannot care about it due to my lack of skills,*’ and another student wrote ‘*I learned some skills of interviewing patients, but I could not empathize with patients very well; I felt like I had lied when I said emphasizing words to them*.’ Taken together, these findings from the open-ended responses appear to indicate that students experience difficulties in empathizing with patients emotionally yet do manage to acquire the cognitive skills that are needed to communicate with patients. This interpretation is in line with the confirmatory factor analysis outcomes in that the correlation between compassionate care and willingness to show empathic behaviour was substantially lower than the correlation between perspective taking and willingness to show empathic behaviour.

## Discussion

The current study examined how perspective taking and compassionate care relate to willingness to show empathic behaviour and how these relations may change with communication skills training. Although for both perspective taking and compassionate care the correlation with willingness to show empathic behaviour was a bit higher for post-training than for retrospective pre-training, the correlation between perspective taking and willingness to show empathic behaviour was on both occasions substantially higher than the correlation between compassionate care and willingness to show empathic behaviour. In this light, the open-ended question responses revealed among others a difficulty of feeling compassion despite showing empathic behaviour. These findings shed light on the conceptual structure of empathy among medical students and generate a number of hypotheses for future intervention and longitudinal studies on the relation between communication skills training and empathy.

### Cultural context

Future studies could replicate the current study in a different stage of the medical curriculum and subsequent career as well as in different cultural settings. The associations of medical students’ empathy scores with outcomes such as professionalism, empathic behaviour in later years of the curriculum, or patient satisfaction and compliance may have been studied quite well in Western contexts [16, 28–30] but remain relatively underexplored in non-Western cultures. In the Japanese context, empathy is also considered a critical component of professionalism, especially linked with the Bushido concept of *jin* or benevolence [31]. However, future studies are needed with regard to how Japanese physicians or medical students show empathic behaviour to patients and how this is linked to patient outcomes.

### Curriculum factors

Apart from considering cultural context, some new studies should focus on the longitudinal development of perspective taking, compassionate care and willingness to show empathic behaviour. The current study provided some indication that fourth-year medical students may not be able to care a lot about showing compassion because they lack skills that may be needed to facilitate perspective taking. With their rather preliminary cognitive schemas of the type of cases under consideration, the average fourth-year medical student may need to allocate their working memory resources to cognitive processes that are needed for perspective taking to such an extent that fewer resources are available for dealing with emotion or compassionate care [32, 33]. Although this may sound odd at first, research has demonstrated that emotion in patient encounter may—certainly in the early stages of learning—consume working memory resources that are needed for cognitive processes related to learning [34, 35]. Longitudinal studies could provide more insight into how cognitive and emotive processes affect students’ willingness to show empathic behaviour as they learn and enter subsequent stages of training.

### Assessment effects

Another factor that may influence students’ focus or lack of focus on compassionate care, even when their cognitive schemas are sufficiently developed to engage in compassionate care, is that of the assessment context. There is some evidence that adding a hypothesis-driven component to OSCEs can foster the engagement of clinical reasoning when preparing for an OSCE if students expect that they are going to be assessed on clinical reasoning as well [36]. Future experimental studies could test to what extent such (pre-)assessment effects also occur when adding a compassionate care component to the OSCE assessment. Given the apparent importance of developed cognitive schemas, one might expect an interaction between cognitive schemas and assessment context: the assessment context might not matter that much among students whose cognitive schemas are still limited (e.g., fourth-year medical students), but might well make a difference at a later stage. If so, the interaction might explain why in a previous study fifth-year medical students weighed more on the emotive component of empathy, whereas residents weighed more on the cognitive component of empathy [15]. If the students in that study expected to be assessed predominantly on the cognitive component, any focus on the emotive component might be extraneous activity in the context of their preparation for that assessment.

### Different approaches to training

The training in the current study has been a regular part of the medical curriculum in the universities that participated in this study and, as in the vast majority of cases, did not undergo additional validation. Future studies could consider alternative approaches to training such as the Process Communication Model ® [37, 38]. Based on motivational and personality theory, this model provides an approach to teaching communication and training communication skills.

### Measurement and triangulation

Finally, our willingness to show empathic behaviour items did not undergo validation additional to the establishment of face validity. Although the willingness to show empathic behaviour items developed for this study fit in the context of the training of focus in this study and are easy to administer, this set of items could be developed further and adapted for different contexts. Additionally, future studies should consider using direct observation and other alternatives to self-reported willingness to show empathic behaviour.

## Caption Electronic Supplementary Material

Appendix 1. Correlation matrix for both occasions
