# Supplementary material for: Communication skills training and the conceptual structure of empathy among medical students
Source: Perspect Med Educ. 2018 Apr 18;7(4):264–71. doi: 10.1007/s40037-018-0431-z (PMC6086812; doi:10.1007/s40037-018-0431-z)
Supplement: Supplementary file 1 — Appendix 1. Correlation matrix for both occasions [file 40037_2018_431_MOESM1_ESM.docx]

Appendix 1. Correlation matrix for both occasions.

| **Correlation Matrix - Retrospective Pre** | | | | | | | | | | | | | | | | | | | | | | | |
| --- | --- | --- | --- | --- | --- | --- | --- | --- | --- | --- | --- | --- | --- | --- | --- | --- | --- | --- | --- | --- | --- | --- | --- |
|  |  |  |  |  |  |  |  |  |  |  |  |  |  |  |  |  |  |  |  |  |  |  |  |
| **Item** | **1** | **2** | **3** | **4** | **5** | **6** | **7** | **8** | **9** | **10** | **11** | **12** | **13** | **14** | **15** | **16** | **17** | **18** | **19** | **20** | **21** | **22** | **23** |
| **1** | — | 0.143 | 0.120 | 0.156 | -0.027 | 0.099 | 0.387 | 0.439 | 0.116 | 0.045 | 0.391 | 0.377 | 0.132 | 0.350 | 0.132 | 0.193 | 0.016 | 0.043 | 0.269 | 0.211 | 0.059 | 0.067 | 0.035 |
| **2** |  | — | -0.159 | 0.383 | 0.274 | -0.063 | 0.212 | 0.244 | 0.394 | 0.245 | 0.185 | 0.176 | 0.421 | 0.207 | 0.251 | 0.364 | 0.228 | 0.012 | 0.102 | 0.363 | 0.298 | 0.339 | 0.330 |
| **3** |  |  | — | -0.174 | -0.090 | 0.426 | -0.076 | 0.025 | -0.147 | -0.140 | 0.008 | 0.023 | -0.139 | 0.090 | -0.151 | -0.191 | -0.121 | 0.049 | 0.017 | -0.113 | -0.170 | -0.177 | -0.179 |
| **4** |  |  |  | — | 0.353 | -0.169 | 0.199 | 0.259 | 0.512 | 0.339 | 0.186 | 0.297 | 0.516 | 0.162 | 0.276 | 0.505 | 0.242 | -0.055 | 0.171 | 0.367 | 0.275 | 0.330 | 0.312 |
| **5** |  |  |  |  | — | -0.115 | -0.047 | 0.018 | 0.199 | 0.225 | 0.014 | -0.015 | 0.291 | -0.026 | 0.096 | 0.133 | 0.165 | 0.026 | -0.042 | 0.263 | 0.240 | 0.231 | 0.213 |
| **6** |  |  |  |  |  | — | 0.089 | 0.143 | -0.117 | -0.131 | 0.172 | 0.095 | -0.102 | 0.172 | -0.181 | -0.074 | -0.108 | 0.118 | 0.076 | -0.077 | -0.141 | -0.145 | -0.126 |
| **7** |  |  |  |  |  |  | — | 0.728 | 0.255 | 0.148 | 0.568 | 0.610 | 0.278 | 0.474 | 0.186 | 0.386 | 0.147 | 0.082 | 0.405 | 0.302 | 0.145 | 0.161 | 0.162 |
| **8** |  |  |  |  |  |  |  | — | 0.318 | 0.202 | 0.671 | 0.642 | 0.351 | 0.567 | 0.169 | 0.435 | 0.159 | 0.156 | 0.407 | 0.352 | 0.192 | 0.210 | 0.210 |
| **9** |  |  |  |  |  |  |  |  | — | 0.351 | 0.245 | 0.332 | 0.577 | 0.256 | 0.297 | 0.654 | 0.316 | -0.056 | 0.176 | 0.459 | 0.400 | 0.412 | 0.448 |
| **10** |  |  |  |  |  |  |  |  |  | — | 0.222 | 0.138 | 0.406 | 0.085 | 0.269 | 0.315 | 0.205 | 0.043 | -0.009 | 0.316 | 0.266 | 0.252 | 0.263 |
| **11** |  |  |  |  |  |  |  |  |  |  | — | 0.648 | 0.290 | 0.596 | 0.094 | 0.371 | 0.105 | 0.194 | 0.451 | 0.314 | 0.206 | 0.246 | 0.232 |
| **12** |  |  |  |  |  |  |  |  |  |  |  | — | 0.343 | 0.477 | 0.127 | 0.418 | 0.099 | 0.130 | 0.461 | 0.291 | 0.173 | 0.203 | 0.199 |
| **13** |  |  |  |  |  |  |  |  |  |  |  |  | — | 0.290 | 0.330 | 0.602 | 0.350 | -0.032 | 0.136 | 0.534 | 0.477 | 0.477 | 0.483 |
| **14** |  |  |  |  |  |  |  |  |  |  |  |  |  | — | 0.129 | 0.326 | 0.085 | 0.244 | 0.394 | 0.343 | 0.243 | 0.249 | 0.233 |
| **15** |  |  |  |  |  |  |  |  |  |  |  |  |  |  | — | 0.424 | 0.310 | -0.073 | 0.024 | 0.467 | 0.272 | 0.244 | 0.266 |
| **16** |  |  |  |  |  |  |  |  |  |  |  |  |  |  |  | — | 0.360 | -0.114 | 0.303 | 0.554 | 0.472 | 0.487 | 0.495 |
| **17** |  |  |  |  |  |  |  |  |  |  |  |  |  |  |  |  | — | -0.127 | -0.010 | 0.284 | 0.270 | 0.262 | 0.263 |
| **18** |  |  |  |  |  |  |  |  |  |  |  |  |  |  |  |  |  | — | 0.037 | 0.083 | -0.018 | -0.036 | -0.004 |
| **19** |  |  |  |  |  |  |  |  |  |  |  |  |  |  |  |  |  |  | — | 0.201 | 0.130 | 0.178 | 0.160 |
| **20** |  |  |  |  |  |  |  |  |  |  |  |  |  |  |  |  |  |  |  | — | 0.599 | 0.576 | 0.581 |
| **21** |  |  |  |  |  |  |  |  |  |  |  |  |  |  |  |  |  |  |  |  | — | 0.947 | 0.904 |
| **22** |  |  |  |  |  |  |  |  |  |  |  |  |  |  |  |  |  |  |  |  |  | — | 0.934 |
| **23** |  |  |  |  |  |  |  |  |  |  |  |  |  |  |  |  |  |  |  |  |  |  | — |
| **Correlation Matrix – Post** | | | | | | | | | | | | | | | | | | | | | | | |
|  |  |  |  |  |  |  |  |  |  |  |  |  |  |  |  |  |  |  |  |  |  |  |  |
| **Item** | **1** | **2** | **3** | **4** | **5** | **6** | **7** | **8** | **9** | **10** | **11** | **12** | **13** | **14** | **15** | **16** | **17** | **18** | **19** | **20** | **21** | **22** | **23** |
| **1** | — | 0.083 | 0.062 | 0.091 | -0.053 | 0.188 | 0.389 | 0.388 | 0.046 | 0.050 | 0.358 | 0.319 | 0.117 | 0.348 | 0.070 | 0.113 | -0.004 | 0.027 | 0.261 | 0.128 | 0.081 | 0.086 | 0.046 |
| **2** |  | — | -0.186 | 0.366 | 0.251 | -0.051 | 0.211 | 0.224 | 0.362 | 0.274 | 0.252 | 0.210 | 0.334 | 0.220 | 0.197 | 0.351 | 0.246 | 0.047 | 0.091 | 0.347 | 0.284 | 0.291 | 0.298 |
| **3** |  |  | — | -0.204 | -0.142 | 0.526 | -0.006 | -0.005 | -0.199 | -0.098 | -0.009 | -0.094 | -0.150 | 0.034 | -0.143 | -0.217 | -0.035 | 0.031 | -0.050 | -0.224 | -0.176 | -0.201 | -0.180 |
| **4** |  |  |  | — | 0.334 | -0.103 | 0.220 | 0.244 | 0.469 | 0.371 | 0.237 | 0.230 | 0.483 | 0.205 | 0.251 | 0.504 | 0.193 | 0.035 | 0.101 | 0.426 | 0.353 | 0.383 | 0.383 |
| **5** |  |  |  |  | — | -0.130 | -0.004 | -0.035 | 0.158 | 0.164 | -0.026 | 0.000 | 0.266 | -0.074 | 0.070 | 0.131 | 0.142 | -0.025 | -0.028 | 0.238 | 0.283 | 0.272 | 0.280 |
| **6** |  |  |  |  |  | — | 0.140 | 0.156 | -0.100 | -0.125 | 0.150 | 0.149 | -0.091 | 0.138 | -0.167 | -0.076 | -0.043 | 0.100 | 0.066 | -0.099 | -0.068 | -0.105 | -0.101 |
| **7** |  |  |  |  |  |  | — | 0.720 | 0.224 | 0.078 | 0.533 | 0.501 | 0.306 | 0.489 | 0.080 | 0.332 | 0.089 | 0.148 | 0.368 | 0.168 | 0.209 | 0.186 | 0.163 |
| **8** |  |  |  |  |  |  |  | — | 0.271 | 0.150 | 0.600 | 0.562 | 0.286 | 0.579 | 0.092 | 0.425 | 0.090 | 0.231 | 0.369 | 0.240 | 0.229 | 0.224 | 0.235 |
| **9** |  |  |  |  |  |  |  |  | — | 0.327 | 0.226 | 0.196 | 0.489 | 0.220 | 0.224 | 0.580 | 0.250 | 0.052 | 0.133 | 0.410 | 0.407 | 0.374 | 0.393 |
| **10** |  |  |  |  |  |  |  |  |  | — | 0.177 | 0.062 | 0.351 | 0.067 | 0.301 | 0.289 | 0.264 | 0.045 | 0.020 | 0.361 | 0.283 | 0.229 | 0.269 |
| **11** |  |  |  |  |  |  |  |  |  |  | — | 0.667 | 0.349 | 0.705 | 0.056 | 0.394 | 0.075 | 0.230 | 0.462 | 0.394 | 0.294 | 0.288 | 0.281 |
| **12** |  |  |  |  |  |  |  |  |  |  |  | — | 0.295 | 0.541 | 0.005 | 0.343 | 0.000 | 0.166 | 0.532 | 0.271 | 0.272 | 0.295 | 0.275 |
| **13** |  |  |  |  |  |  |  |  |  |  |  |  | — | 0.291 | 0.293 | 0.536 | 0.370 | -0.021 | 0.169 | 0.475 | 0.511 | 0.527 | 0.547 |
| **14** |  |  |  |  |  |  |  |  |  |  |  |  |  | — | 0.083 | 0.322 | 0.022 | 0.235 | 0.418 | 0.330 | 0.286 | 0.285 | 0.289 |
| **15** |  |  |  |  |  |  |  |  |  |  |  |  |  |  | — | 0.302 | 0.257 | -0.048 | -0.006 | 0.363 | 0.197 | 0.177 | 0.207 |
| **16** |  |  |  |  |  |  |  |  |  |  |  |  |  |  |  | — | 0.242 | -0.057 | 0.269 | 0.546 | 0.490 | 0.470 | 0.453 |
| **17** |  |  |  |  |  |  |  |  |  |  |  |  |  |  |  |  | — | -0.087 | -0.025 | 0.276 | 0.313 | 0.311 | 0.328 |
| **18** |  |  |  |  |  |  |  |  |  |  |  |  |  |  |  |  |  | — | 0.032 | 0.045 | 0.116 | 0.081 | 0.140 |
| **19** |  |  |  |  |  |  |  |  |  |  |  |  |  |  |  |  |  |  | — | 0.214 | 0.125 | 0.144 | 0.168 |
| **20** |  |  |  |  |  |  |  |  |  |  |  |  |  |  |  |  |  |  |  | — | 0.623 | 0.608 | 0.615 |
| **21** |  |  |  |  |  |  |  |  |  |  |  |  |  |  |  |  |  |  |  |  | — | 0.924 | 0.889 |
| **22** |  |  |  |  |  |  |  |  |  |  |  |  |  |  |  |  |  |  |  |  |  | — | 0.916 |
| **23** |  |  |  |  |  |  |  |  |  |  |  |  |  |  |  |  |  |  |  |  |  |  | — |
|  | | | | | | | | | | | | | | | | | | | | | | | |
